# Recent advances in the inference of deep viral evolutionary history

**DOI:** 10.1128/jvi.00292-25

**Published:** 2025-08-22

**Authors:** Jonathon C. O. Mifsud, Marc A. Suchard, Edward C. Holmes, Philippe Lemey

**Affiliations:** 1 School of Medical Sciences, The University of Sydney 216920, Sydney, New South Wales, Australia; 2 Department of Microbiology, Immunology and Transplantation, Rega Institute, KU Leuven 573654 , Leuven, Belgium; 3 Department of Biomathematics, David Geffen School of Medicine at UCLA, University of California 207035, Los Angeles, California, USA; 4 Department of Biostatistics, Jonathan and Karin Fielding School of Public Health, University of California 309948, Los Angeles, California, USA; 5 Department of Human Genetics, David Geffen School of Medicine at UCLA, University of California 196265, Los Angeles, California, USA; Universiteit Gent, Merelbeke, Belgium

**Keywords:** virus evolution, structural phylogenetics, time-dependent rates, substitution saturation, 3Di alphabet

## Abstract

The rapid rate of virus evolution, while useful for outbreak investigations, poses a challenge for accurately estimating long-term viral evolutionary divergence and leaves us with little genomic traces at deep evolutionary timescales, complicating the reconstruction of deep virus evolutionary history. Recent advancements in protein structure prediction and computational biology have opened up new avenues and enabled us to peer back further in time and with greater clarity than ever before. Here, we review recent approaches to reconstructing the deep evolutionary history of viruses. In particular, we focus on how Bayesian models that account for evolutionary rates that are time-dependent may provide better estimates of the timescale of virus evolution. We then outline approaches to structural phylogenetics and their application to reconstructing the evolutionary history of viruses. Despite current limitations, including structural prediction uncertainty, conformational variation, and limited benchmarking, structural phylogenetics appears promising, particularly where sequence-level homology is eroded. The availability of and ease with which virus structures can now be predicted is likely to drive additional statistical and software developments in this area. Ultimately, answering fundamental questions of virus origins and early diversification, long-term host associations, virus classification, and the timescale of viral diseases will likely require unifying sequence and structural information into a temporally aware evolutionary inference framework.

## INTRODUCTION

Phylogenetic studies examining the origins, emergence, and spread of viruses have arguably been one of the most active and successful areas of evolutionary biology and form the bedrock of the flourishing field of genomic epidemiology. This, in part, reflects the ability of viruses, particularly those with RNA genomes, to evolve at rates much greater than their cellular counterparts (1). The rapid rate at which viruses evolve and accumulate mutations enables evolutionary signals to be identified through comparative genomics at short timescales relevant for outbreak investigation and response. The integration of phylogenetics and epidemiology, known as phylodynamics, has become a vital tool in response to numerous viral outbreaks, epidemics, and pandemics, including Ebola (2), Zika (3), and, more recently, COVID-19 (4) and mpox (5).

**Fig 1:**
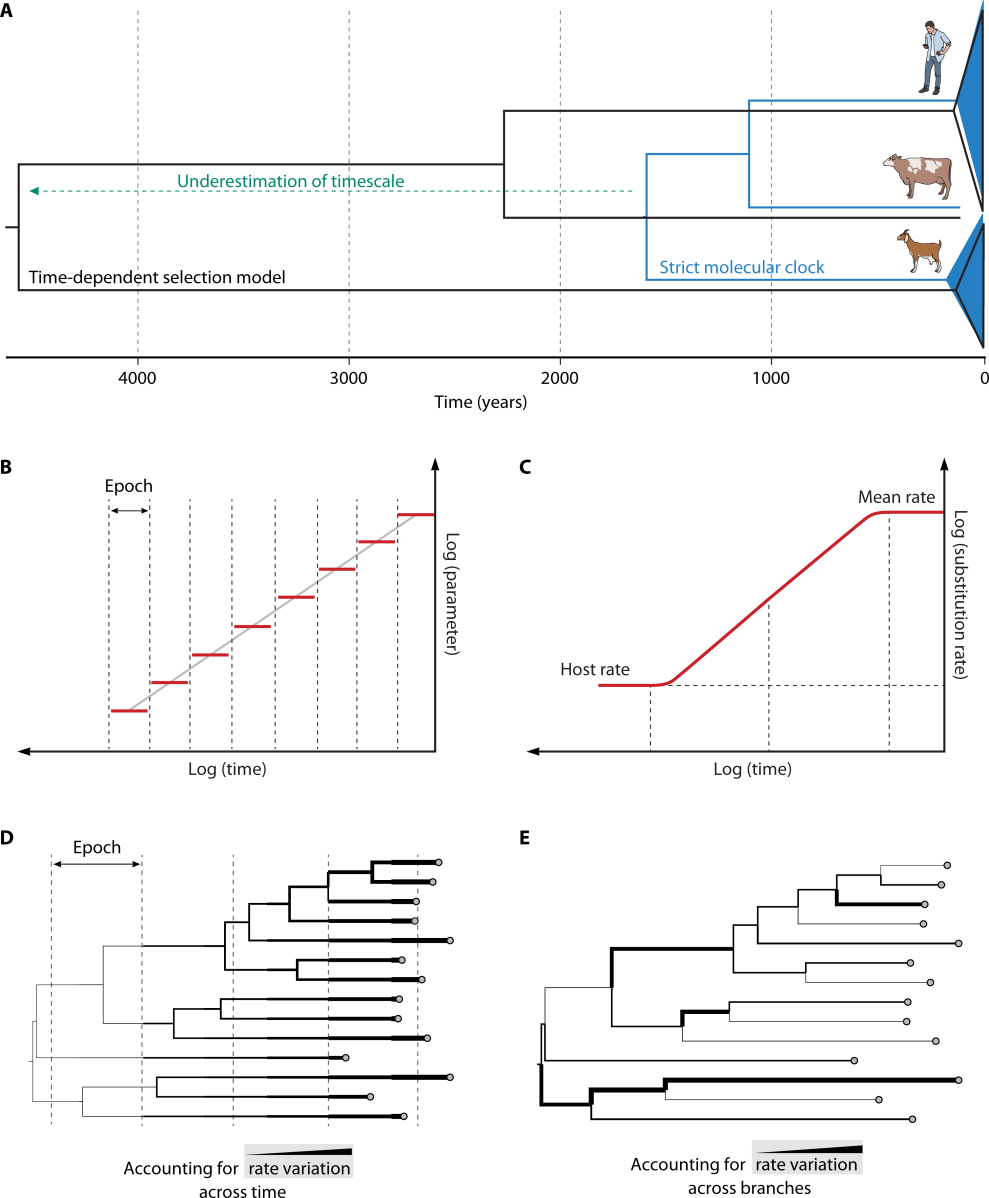
Modeling time-dependent selection and evolutionary rates. (**A**) Comparison of the timescale for measles, rinderpest, and peste des petits ruminants virus under a strict molecular clock represented by a blue phylogeny and under a time-dependent selection model represented by black phylogeny branches. The differences between the two time-scale estimates are highlighted in green. (**B**) Conceptual representation of modeling variability in evolutionary parameters through time. A piecewise constant model for parameter changes through time is represented that relies on a linear relationship between log(parameter) and log(rate) (gray line) across different time intervals; the parameter can be the evolutionary rate or the nonsynonymous/synonymous substitution rate ratio. Splitting the phylogeny into time slices, each with its model (known as epoch modeling), allows for flexible modeling of parameters under arbitrary epoch structures. (**C**) Conceptual representation of the Prisoner-of-war model that models a rate decline when sites start to experience nucleotide substitution saturation.(**D**) Rate variation through time as modeled using the epoch TDR approach (in B). Rate variation is represented by differences in branch thickness. (**E**).Rate variation among branches as modeled using uncorrelated relaxed clock models (15).

The same propensity for viruses to evolve rapidly also leads to the erosion of evolutionary signals over deep timescales, defined here as occurring more than 1,000 years ago. In particular, short-term evolutionary dynamics cannot be directly extrapolated to deep timescales (6, 7), such that evolutionary rates in RNA viruses can be considered to be “time-dependent” (8, 9). As standard evolutionary models start to fail in adequately correcting for multiple substitutions at single sites, sequence divergence will increasingly be underestimated over larger timescales. While phylogenetic tree topologies may still be adequately inferred under such circumstances, their timescales will be poorly estimated. For example, this has been demonstrated for measles virus, which was first estimated to have diverged from rinderpest a little over 1,000 years ago (10), but revised to about the sixth century BCE using models that account for long-term purifying selection (11) (Fig. 1A). Importantly, under long-term purifying selection (12), multiple substitutions even leading to complete mutational saturation at synonymous sites may remain hidden in sequences that are relatively conserved at the amino acid level. Over timescales of millions of years, this may result in dramatic time-dependent rates (TDR), as illustrated for the foamy viruses and primate immunodeficiency viruses (13, 14). These highlight clear temporal boundaries to how far back we can reliably reconstruct deep virus evolutionary history using extant sequence data.

The challenge of accurately estimating viral evolutionary rates and the limited genomic traces we are left with at deep timescales means that we remain in the dark when it comes to many of the fundamental questions in virus evolution, such as the origins of virus lineages, virus-host coevolutionary relationships, and the evolution of viral complexity. Looking forward, however, the recent revolution in artificial intelligence (AI) technology and its biological applications (e.g., AlphaFold2), as well as the development of more sophisticated phylogenetic methods and models of virus evolution which account for the complexities introduced when working at deep timescales, offer new opportunities to study the deep evolutionary history of viruses. In this minireview, we briefly discuss the challenges and longstanding questions in virus evolution and highlight how recent technological advancements present new avenues to explore these questions.

## ACCOMMODATING TIME-DEPENDENT EVOLUTIONARY RATES

The apparent decline in evolutionary rate over deep timescales is now well established in viruses (6, 8, 9, 16). The decline is generally only “apparent” as it reflects challenges in recovering evolutionary divergence over deep timescales. Adequately modeling long-term purifying selection may allow estimating divergence times for rapidly evolving viruses over timescales of 1,000s of years (11) (Fig. 1A), but for deeper evolutionary histories, substitution saturation will prevent the recovery of substitution histories. For example, foamy viruses (*Retroviridae*) have co-diverged with their primate hosts for over a hundred million years. However, virus evolutionary rates inferred over this time period are ~4–5 orders of magnitude lower than those observed in the short term (13). Because such dramatic rate decays cannot be accounted for by substitution models, phenomenological correction through the molecular clock model has been proposed (14). Specifically, this motivated the development of formal TDR models that allow for rate variation through time in a Bayesian framework. The Bayesian approach models a relationship between rate and time throughout evolutionary history (Fig. 1B and D) and allows incorporating different sources of calibration (both internal node calibration and dated tip calibration) to estimate how rapidly the rate declines in time as well as the timescale over which this occurred (Fig. 1B and D). Currently, no additional sources of evolutionary rate variation are considered, which can limit the applicability of TDR models. For example, a study of hepatitis B virus (HBV) ancient genomes indicated that a TDR model could estimate HBV divergence times more consistently with ancient human migration events compared to estimates under an uncorrelated relaxed clock model (17). However, the model offered a poor fit to the data because it was unable to account for considerable rate variation among branches (represented in Fig. 1E).

Building on the observation of a seemingly consistent decline in evolutionary rate over time across different virus groups (9), an alternative model has also been proposed that explains the rate decay as a dynamic process of substitution saturation across sites evolving at different rates. In this model, sites can begin to saturate after a few decades or hundreds of years, at which point the rate starts to decline, eventually converging with the host evolutionary rate. Because this model is inspired by the idea that viral sequence space is relatively small and restrictive, it has been termed the “Prisoner of War” (PoW) model (18, 19). To correct for substitution saturation, this approach applies a transformation to a phylogeny estimated using a standard nucleotide substitution model. While the PoW model and the Bayesian TDR approach have proven useful in different scenarios (20–22), further development could potentially converge toward a PoW-style parameterization within the Bayesian phylogenetic framework that can accommodate other sources of evolutionary rate variation in addition to rate variation through time and use different molecular clock calibrations.

## EROSION OF EVOLUTIONARY SIGNAL IN VIRUSES

While nucleotide sequences are commonly the choice when comparing viruses over outbreak timescales, the small size of the DNA/RNA alphabet has disadvantages for sequences that have diverged over thousands to millions of years. Indeed, randomly aligned nucleotide sequences are expected to have 25% identity by chance alone, even greater if gaps are permitted. Given the much larger size of the amino acid alphabet and the ability to model different rates of amino acid substitutions, they have been commonly used to determine the phylogenetic relationships among distantly related virus sequences. However, as with nucleotide sequences, as we go deeper into evolutionary time, little amino acid conservation is likely to remain across even the most conserved regions of the virus genome, including the RNA-dependent RNA polymerase (RdRp). For example, in efforts to construct an alignment from across the RNA virus diversity (23), the pairwise identity between sequences was often extremely low, with a mean value of 7.7% and a minimum of 1% (24). This approaches the level of pairwise identity expected by chance alone (5%). Here, evolutionary signal is likely to be obscured by high levels of sequence divergence and compounded by site saturation, leading to substantial underestimation of divergence and increased phylogenetic error. In some cases, multiple sequence alignment (MSA) bias and error—which are likely to increase with genetic divergence—can result in high confidence (bootstrap support) for incorrect topologies (25). As a result, it not only becomes difficult to infer the timescales of virus evolution, but also the broader phylogenetic and taxonomic relationships among viruses may be distorted.

## INCORPORATING PROTEIN STRUCTURE

As protein structure is estimated to be 3–10 times more conserved than amino acids alone (26) and is a powerful way of inferring homology between highly divergent sequences (27, 28), researchers have long considered incorporating structural information into phylogenetic reconstructions of viruses. The earliest known application of this method was by Chelvanayagam, Heringa, and Argos (29), who constructed dendrograms based on the distances between C_α_ atoms obtained through the superpositions of 25 viral capsid structures. Importantly, they noted that the structural dendrograms resulted in a similar topology to those generated using sequence data. In the following years, the incorporation of structural information into studies of virus evolution involved tools such as MUSTANG (30), FATCAT (31), Structure Homology Program (SHP) (32), and later the Homologous Structure Finder (HSF) (33) (reviewed in reference 34). These approaches inferred phylogenies using the Fitch-Margoliash criterion, which takes distances generated from pairwise alignments—here using protein structures—and evaluates all possible trees to find the one that minimizes the squared differences between observed pairwise distances and the distances predicted by the tree. Pairwise alignments can then be supplemented with information derived from aspects of secondary structure and physicochemical and geometrical properties to increase the likelihood of residue equivalence between structures (34). Another approach was to generate structurally informed sequence alignments as implemented in tools such as EXPRESSO (35) and MAFFT-DASH (36). This involved integrating tertiary structural information and constraints into amino acid MSA scoring through precomputed structural alignments. An advantage of this approach is that the resulting alignments remain in amino acid format, enabling the use of sophisticated models of sequence evolution in a maximum likelihood (ML) framework (37, 38) or Bayesian inference frameworks (39). These frameworks have a number of advantages in that they allow for the selection of models of sequence evolution and provide measures of statistical uncertainty in tree topology through bootstrapping (ML) or posterior probabilities (Bayesian inference). Biochemical and structural features (e.g., replication mode, polymerase core organization, motif lengths, and conformations) have also been encoded in character states–akin to how morphological trait data are incorporated–and used alongside amino acids in Bayesian phylogenetics (40).

Despite its promise, however, the application of structural phylogenetics in evolutionary virology has been limited (40–64). Indeed, recent efforts to infer the timing and origins of pathogen emergence in humans (11, 65, 66), construct a universal RNA virus phylogeny (67), and establish high-level taxonomy structures (23, 68) have all relied solely on sequence data. Likewise, there has been limited development of new software and little formal integration of structural information into core software packages used by the field (e.g., IQTREE [69] and BEAST [70]). A key limiting factor has long been the absence of sufficient, high-quality protein structures, as their generation required labor-intensive and expensive techniques, such as X-ray crystallography, nuclear magnetic resonance, and cryogenic electron microscopy. However, with the recent advancements in computational protein structure prediction (e.g., AlphaFold2 and 3 [71, 72], RoseTTAFold [73], and ESMFold [74]), this is rapidly being overcome. In just the past year, the structures of over 500,000 virus proteins have been predicted (75–78). Given ongoing improvements and attention directed toward these tools (72, 79), it is clear that the number and quality of these predicted structures will continue to increase.

## THE PROMISE OF STRUCTURAL CHARACTERS FOR UNDERSTANDING VIRUS EVOLUTION

**Fig 2:**
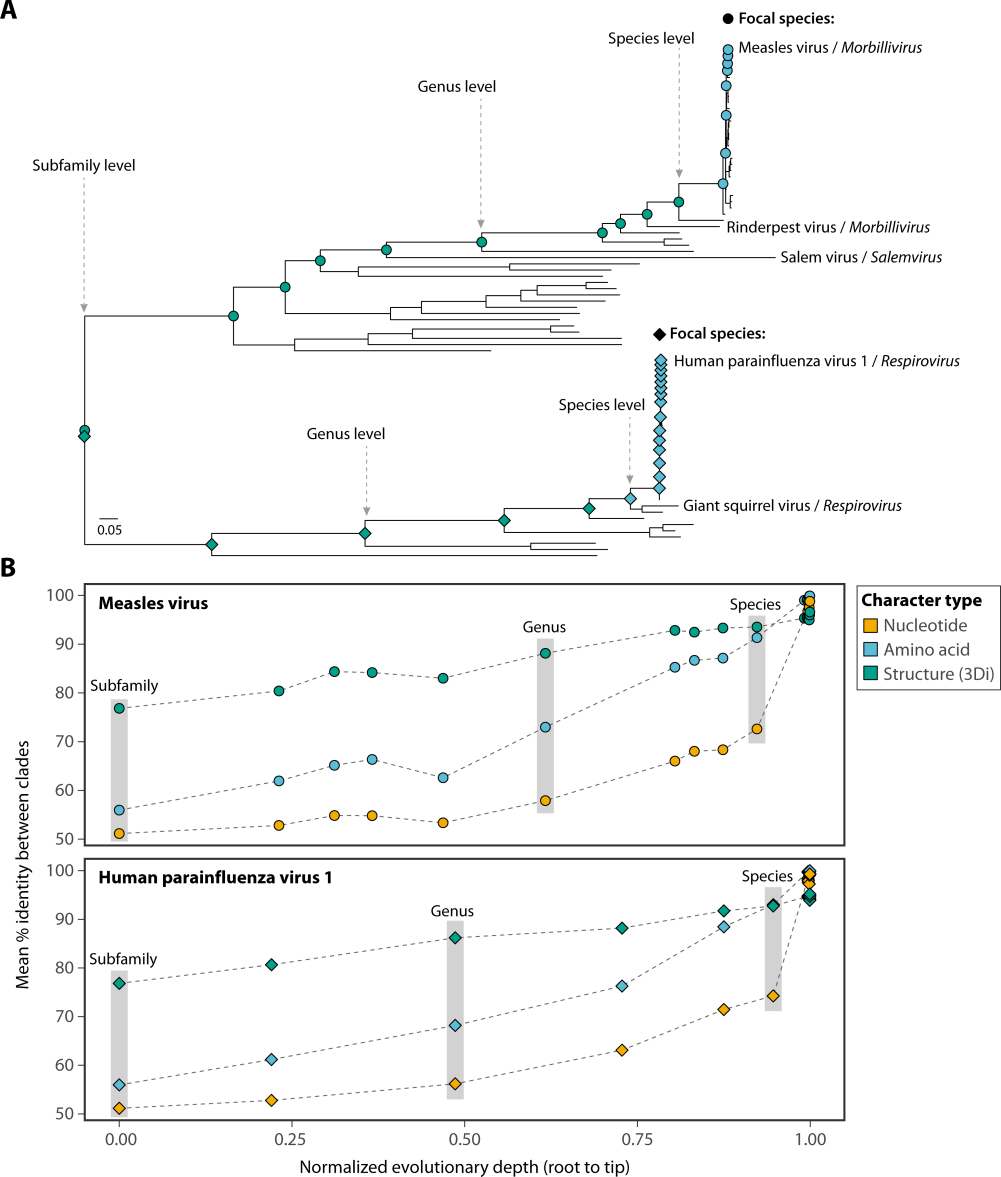
Comparison of phylogenetic character identity across the evolutionary depth of the *Orthoparamyxovirinae*. (**A**) Maximum likelihood phylogeny of representative *Orthoparamyxovirinae* L protein sequences rooted on the Wēnlǐng triplecross lizardfish paramyxovirus (*Synodonvirus*) (not shown). Node symbols show the path from each focal virus (measles virus and human parainfluenza virus 1) to the root. Node symbols are colored by character type with the highest mean identity at that node (structure/3Di [teal], amino acid [blue], or nucleotide [amber]). Symbol shape distinguishes focal viruses: circles for measles virus (*Morbillivirus*), diamonds for human parainfluenza virus 1 (*Respirovirus*). ICTV taxonomic information is annotated to demonstrate, for example, at the “genus” level where the focal virus, measles, and members from another genus meet. Scale bar denotes the number of amino acid substitutions per site (**B**). Mean identity decay curves for each focal virus. For each internal node from tip to root, the mean pairwise identity between the two immediate child clades is calculated. Taxonomic levels are annotated as in panel A. Identity values are derived from global pairwise alignments. For 3Di comparisons, the custom 3Di substitution matrix from (82) was used.

Extracting evolutionary information from the vast number of protein structures has also proved challenging computationally. Structural alignment and downstream phylogenetics, particularly those that work directly with 3D structural information (e.g., DALI [80] and TM-align [81]), require numerous pairwise structural alignments, making core tasks such as homolog searching and building multiple structural alignments for phylogenetics computationally intensive. For example, it requires a month of computing time on a single CPU core to compare a single protein structure against a protein structure database of 100 million structures using TM-align (81, 82). One potential solution taken by the software FoldSeek (82) is to reduce structures to lower-dimensional character-state representations of the interactions between each residue—specifically the C_α_—in the structure and its nearest neighbors by Euclidean distance. In doing so, these characters, termed 3Di, capture elements of tertiary structure and reduce structural alignments to much more computationally efficient character alignments. 3Di character prediction is done using an unsupervised machine learning model trained on structures from the SCOPe40 structural database (83). FoldSeek makes use of this structural alphabet alongside a 3Di substitution matrix to search for hits to a query against a database of structures (82). Conveniently, 3Di characters have the same dimensionality as amino acids (*n* = 20) and seem to retain the signal of the structural folds, leading to the suggestion that these characters could be useful in phylogenetics and incorporated into traditional amino acid-based likelihood methods (46, 84). Indeed, when comparing pairwise identity shared between clades of the *Orthoparamyxovirinae* phylogeny, we observed that 3Di characters were the most conserved characters when clades containing two or more virus species were compared (e.g., measles virus and rinderpest virus) (Fig. 2A and B). At this point, a notable drop in mean percentage identity was seen for nucleotide sequences, while amino acid identity declined toward where comparisons were being made between two genera of the *Orthoparamyxovirinae* (e.g., *Morbillivirus* and *Salemvirus*). When comparing members of the two major clades that make up the *Orthoparamyxovirinae* subfamily, mean percent identity was higher for 3Di (78%) compared to amino acid (56%) and nucleotide characters (51%). The clear plateau in mean nucleotide percent identity highlights the point at which the nucleotide signal is being obscured by sequence saturation.

Recently, several attempts have been made to incorporate 3Di characters into phylogenetics. One approach, packaged as the FoldTree program (46), makes use of various scores of structural change calculated or taken directly from FoldSeek’s all vs all character-based structural alignments and uses these to infer neighbor-joining (NJ) trees. While benchmarking phylogenetic methods is notoriously difficult, based on measures of taxonomic congruence and ultrametricity (a measure of how uniform root-to-tip lengths are for all tree tips, which may reflect adherence to a molecular clock), NJ trees inferred from distances derived from the fraction of identical residues across the 3Di and amino acid-based alignments, known as Fident, outperformed amino acid-based approaches (46). Notably, the structurally informed trees were consistently better across small and large protein family evolutionary distances, suggesting that the incorporation of structural-based information in phylogenetics may be useful for investigations across differing evolutionary scales (46). Using this approach, Moi et al. (46) investigated the RRNPPA family of rapidly evolving cytosolic communication receptors and, for the first time, were able to reconstruct a phylogeny across all of the diverse subfamilies, including those found across *Firmicutes* bacteria, their conjugative elements, and viruses.

A second approach is to incorporate 3Di characters directly into amino acid MSA programs and interpret them as conventional phylogenetic characters (84). Consistent with the advantages seen when used in homology detection, MSAs constructed from the 3Di characters of the ferritin-like superfamily of proteins showed substantially more conservation (116 sites are over 50% conserved compared to 17 sites for amino acids) when comparing inter-family proteins in the twilight zone (i.e., those sharing 20%–30% amino acid similarity) (84). 3Di also appeared to capture structurally conserved signals that are missed when using amino acids (84). Here, the amino acid and 3Di alignments were combined under a partition model, each using the best-fitting substitution model (including a custom 3Di-specific substitution model) corresponding to that alignment. The addition of 3Di characters in this manner, termed 3DiPhy, was more resistant to sequence saturation on deep branches and appeared to ameliorate long-branch attraction artifacts, where distantly related lineages occupying long branches are incorrectly grouped together (84). This approach may overcome some of the limitations of distance-based approaches and allow for the implementation of ML phylogenetic estimation and bootstrapping methods.

With the ease with which virus structure can now be predicted and the availability of virus structure databases, approaches such as FoldTree and 3DiPhy offer a promising avenue for the study of deep viral evolutionary history. When applied to the *Flaviviridae*, structure-based prediction and homology searches were more sensitive than amino acid-based methods, allowing for the comparisons of the families’ highly divergent glycoprotein structures (47, 85). In joint 3Di-amino acid phylogenies, 3Di consistently made a greater contribution to phylogenetic inference, and in some cases, clear evidence of long branch attraction present in the amino acid phylogenies and carried over to the joint 3Di-amino acid phylogenies was absent in the 3Di-based phylogenies alone. With the development of programs that leverage 3Di, such as the multiple structural alignment FoldMason (86) and core gene phylogenetic reconstruction software Unicore (87), this approach is now widely accessible and seeing applications in this field (44, 45). In addition, 3Di characters have recently been implemented in Bayesian inference frameworks (88). This framework will be particularly suited to handling the increased uncertainty associated with structural and 3Di character prediction. A Bayesian framework would also offer considerable modeling flexibility across partitions in joint amino acid 3Di inference. In addition, the incorporation of 3Di characters into BEAST would allow for the inference of structural trees in time units, theoretically allowing for the use of a range of molecular clock models. Notably, both 3Di approaches (FoldTree and 3DiPhy) appear to have a stronger clock-like signal than standard maximum likelihood based on amino acid alignments (89). However, this may not be the case for all proteins. For those composed of a high proportion of alpha-helices, the repetitive nature of these regions may lead to 3Di being dominated by a small number of character states (88). In one such study examining histones, 3Di characters were found to have a less clock-like rate of evolution than the amino acids. It is not yet clear which virus proteins are best suited for 3Di character phylogenetics (88).

## PROBABILISTIC MODELS OF VIRUS STRUCTURAL EVOLUTION

To date, viral phylogenetic analysis using structure has primarily relied on structural distances, structure-guided or constrained amino acid alignments, or more recently, character-based encoding (e.g., 3DiPhy). While these approaches can capture evolutionary informative signals through abstracting structural features, they do not explicitly model structural evolution. An alternative is to treat protein structure as a continuous trait and model a diffusion process of either alpha carbon (C_α_) three-dimensional coordinates or dihedral angles for each amino acid (90–96). Through modeling the evolution of structure itself, rather than a derived representation, we gain access to additional information regarding viral protein mechanisms and functional evolution. Notably, unlike structurally informed amino acid and character-based approaches, these models could support ancestral structure reconstruction as part of the inference process. However, while efficient algorithms are available to compute multivariate trait likelihood (97, 98), accounting for correlation among the amino acid positions can impose a major computational bottleneck. Similar to modeling sequence evolution, current approaches have, for the most part, assumed independence among amino acid positions (91, 92), but protein structure models accounting for nearest-neighbor site dependence in pairs of structures do exist (94). Benchmarking of such models and potential future extensions against those using 3Di/Foldseek-derived information would be key in determining the tradeoff between the benefits that come with a more holistic model of structure versus the increased computational resources it requires.

## CONSIDERATIONS WITH THE USE OF PROTEIN STRUCTURAL INFORMATION

Despite a long history of applications to studies of virus evolution, it is clear that the structural phylogenetics approach is still very much in its infancy, and as such, there are a number of limitations that need to be considered with current implementations.

### Structural prediction uncertainty

Several measures of structural prediction performance have been used to score the similarity between predicted and experimental structures. AlphaFold provides four: predicted local distance difference test (pLDDT), predicted aligned error (PAE), predicted template modeling (TM) score, as well as ipTM in AlphaFold3. The specific interpretations and use cases for each metric have been reviewed (99). Briefly, LDDT is an alignment-free method, focusing on how well the local atomic geometry matches that of the reference structure, providing a per-residue score ranging from 0 to 100, the latter representing a perfect match. pLDDT is AlphaFold’s internal estimate of this score. Alternatively, TM-score is calculated by taking the mean of the squared distance between two superimposed structures, with values ranging from 0 to 1, where a score of 0.7 suggests that there is a 90% probability that two structures share the fold (100).

Viral protein structures, particularly those that are highly divergent from well-studied and experimentally characterized viruses, have in some cases proven difficult to predict, displaying low confidence values with no strong hits to structures present in reference databases (47, 77). This is reflected in the higher prevalence of structural clusters with only one member (singletons) in virus structure data sets compared to broader collections such as the AlphaFold Database (77). While singletons could represent novel folds, they appear on average to be shorter in the number of residues and have substantially lower structural confidence scores compared to non-singleton clusters (75, 77, 78). pLDDT appears to increase with the presence of related experimental structures in the training data set and with the depth and diversity of the MSA, generally requiring around 30 diverse members (77, 101). While the median ColabFold MSA depth for human and animal virus sequences ratified by the ICTV (i.e., those in Viro3D) appears to be ~100 sequences, this is likely not the case for much of virus diversity, especially non-mammalian-associated viruses, and does not take into account the need for diversity in MSAs (78). Virus structural prediction performance can be improved by (i) incorporating assembled sequencing data to increase MSA depth (77), (ii) increasing the number of recycles in structural prediction (in AlphaFold2) or (iii) the use of MSA-independent methods such as protein language models (e.g., ESM-2 and ESMFold (74) for highly divergent sequences (47, 78). Further metagenomic sequencing and the discovery of diverse virus sequences will be key here.

**Fig 3:**
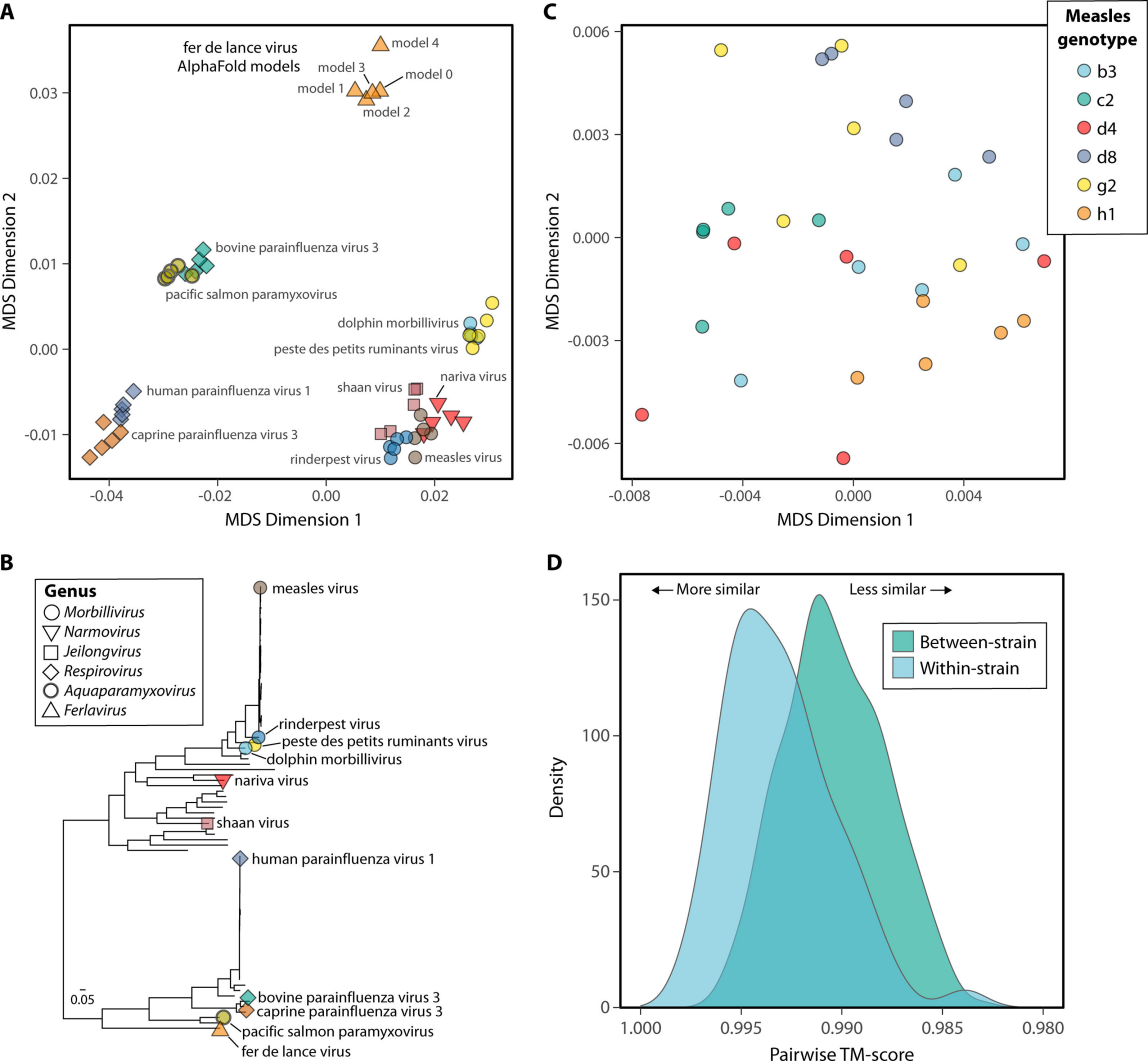
Structural variance between AlphaFold models. (**A**) Multidimensional scaling (MDS) plot of structural dissimilarity derived from pairwise TM-scores for AlphaFold3 predicted L protein structures of select Orthoparamyxovirinae members. Each point represents one of five AlphaFold3 models predicted per virus. Shape varies with virus genus and is colored by species. (**B**) Maximum likelihood phylogeny of representative Orthoparamyxovirinae L protein sequences rooted on the Wēnlǐng triplecross lizardfish paramyxovirus (Synodonvirus) (not shown). Tip labels and shapes are displayed for those viruses present in A. Scale bar denotes the number of amino acid substitutions per site. (**C**) MDS plot as in A, but comparing select measles virus genotypes, with each point representing a single AlphaFold3 model. Colors represent genotypes. (**D**) Density distribution plot of the pairwise TM-scores among measles virus L proteins, split into within-genotypes (i.e., all five AlphaFold3 models for a given sequence) and between measles genotypes.

AlphaFold3 samples protein structures for the same input sequence, templates, and seed five times. The resulting models (0–4) are ranked by average pLDDT, with rank 0 seen as the most reliable. We predicted the L protein structures of select *Orthoparamyxovirinae* members, took each of the five models, and aligned these structures to gauge the structural variation within and between predictions for a given virus species. We observed that models derived from the same sequence, here representing a distinct virus species, tend to cluster together in MDS space (Fig. 3A) and are mostly consistent with phylogenetic groupings from amino-acid-based phylogenies (Fig. 3B). Some clear violations occur, for example, measles and rinderpest virus cluster with nariva virus (*Narmovirus*) and shaan virus (*Jeilongvirus*) away from the other morbilliviruses, dolphin morbillivirus, and peste des petits ruminants virus.

While the benefits of including structural information for highly divergent virus sequences are clear, whether this applies to analyses among strains of a single virus species remains to be seen. At the resolution of virus strains where there is little to no divergence at the amino acid level, structural prediction noise is likely to have an increased effect. Indeed, when comparing measles L protein structure, clear genotype-specific grouping was not observed in MDS analyses (Fig. 3C). Likewise, the distribution of pairwise TM-scores, a measure of agreement between the structures, largely overlapped when comparing within-genotypes (i.e., models 0–4) compared to between genotypes reflecting different underlying sequences (Fig. 3D). This may just reflect the lack of true structural difference between measles genotypes over prediction noise obscuring these relationships, although these sequences do vary to some extent at the amino acid level (97.9% pairwise identity). Structural prediction noise is likely to carry over into the structural characters. Notably, when only considering the top model (model 0), the mean percent identity observed when comparing the L protein of various measles strains was counterintuitively considerably lower for 3Di characters (95.32%) than amino acids (99.02%), closer to that of nucleotide sequences (95.31%) (Fig. 2). Together, this suggests that caution should be taken when conducting structural phylogenetics on closely related virus sequences.

Structural confidence measures such as LDDT appear to significantly influence phylogenetic inference (46). While LDDT has been used to calculate distance-based trees in FoldTree (46), it has largely been ignored in likelihood-based structural phylogenetics approaches beyond its use in filtering out low-confidence structures entirely. LDDT can vary greatly along a protein chain, yet in current implementations, low confidence and disordered regions are typically treated the same as well-conserved and strongly predicted regions, potentially confounding further inferences. In 3Di alignments, it is likely that some phylogenetic error resulting from low-confidence structural prediction can be prevented by trimming columns with a high proportion of gaps (84), or potentially through considering average column-wise LDDT and trimming based on this. Alternatively, LDDT has been implemented as a method of scoring MSAs and to iteratively refine them (86). If multiple models are available for a protein structure, their variability could be encoded in a 3Di sequence as ambiguity codes to take prediction uncertainty into account in phylogenetic inference. As structural similarity is often used as a proxy for homology, similarity derived from prediction error or convergent evolution may be interpreted as shared ancestry. Conversely, missed distant relationships (false negatives) between structures also occur, particularly for low confidence predictions.

### Structural conformers

Even with high-confidence virus structures, one needs to consider that virus proteins can adopt a number of conformations depending on factors such as temperature (102), pH (103), and binding of inhibitors (104). When applied to proteins with more than one experimentally determined conformation, AlphaFold tends to favor one structural conformation–particularly those learned in training–but alternatives can be seen (105, 106). This is particularly problematic for methods that consider rigid body representations of structures, but it may also impact those approaches using intra-molecular distances (107), such as 3Di characters (108), to construct a “worst-case” example of this, demonstrating that 3Di phylogenies can be structured by conformational sampling rather than underlying evolutionary relationships. This problem is likely exacerbated with multi-domain proteins such as the Paramyxoviridae L protein. Here, topological biases resulting from structural conformers might be reduced by splitting the protein into single domains (107).

Methods for exploring structural conformation space are available (109, 110) and have been used to assess the impact on phylogenetic inference. These approaches have proven to be valuable tools for evaluating phylogenetic confidence, although they remain computationally intensive (111). Conformational signal appears to be partially encoded in MSAs, for example, filtering or clustering the MSA input into AlphaFold2, and AlphaFold-multimer can be used to explore alternative conformations (112, 113). This suggests a potential avenue for integrating variation in structural uncertainty and conformations through jointly modeling sequence alignment, structure, and tree topology within a Bayesian framework.

### Convergent structural evolution

An additional concern associated with the use of virus structural phylogenetics is its ability to discern convergent from divergent evolution. Numerous cases of convergent evolution have been observed in viruses, most of which are instances of short-term convergence at a sequence level (114, 115). For example, over half of the substitutions (*n* = 119) in replicate lineages of cultured bacteriophage phiX 174 (5.4kb genome) subjected to similar environmental conditions were observed in other lineages (116). Over larger timescales and when considering protein structure, viral envelopes, capsids, and other immune evasion proteins, each appears to contain examples of convergence (117–120). Given that it is possible for proteins to share nearly identical structures but lack homology at the level of primary sequences, it should not be assumed that similar structures are necessarily homologous. Perhaps reassuringly, a recent search through the FoldSeeks’ clusters of the AlphaFold Database (v4) (112) identified only 2.6% of clusters that lacked sequence-level support for homology (1% was also of high confidence with 80% of positions containing a pLDDT of at least 70 and with strong structure matches, template modeling score ≥0.5). Within this subset, structural repeats were frequently observed. Some originated from sequence tandem repeats, demonstrating inconsistent genealogies relative to the clustered structures. This suggests spurious matches and potentially convergence of structures (121). Currently, knowledge regarding the extent and intensity to which convergence occurs across the virosphere is limited. When examining the presence and likelihood of structural convergence affecting phylogenetic inference, it is important to consider sequence homology, the presence of which would suggest true homology. At levels of genetic divergence where the primary sequence is no longer informative, it is important to consider what regions of the structure share homology and if these are repetitive, while also considering the complexity of the shared homology, assuming that the chance of analogy decreases with shared protein complexity. A number of methods have been developed to detect–and in some cases, quantify–convergent evolution. These include the use of ancestral state reconstruction (122) and analyses of phenotypic similarity by phylogenetic relatedness (123). However, the convergence of viral structures has yet to be fully examined through this lens. Recent advancements to analyze virus functional and structural convergence alongside genetics and how these contribute to adaptive evolution are likely to yield more evolutionary insights.

### 3Di characters

The use of 3Di characters as a proxy of structure has clear advantages for the study of deep viral evolution in its speed and out-of-the-box access to likelihood methods. However, several considerations must also be made. First, it is not clear that current implementations of structural character phylogenetics approaches outperform sequence-based methods (89), particularly at shorter evolutionary distances. There are also clear violations in modeling assumptions such as site independence, and the limited number of substitution models (82, 108) currently available may fall short in exploring the complexities of protein evolution. In addition, it is clear that spatial information is lost when converted to 3Di, given its limited alphabet. It is possible that the complexities of proteins are better captured by more character states or through characterizing structural features in independent alphabets, as implemented in Reseek (124) and Muscle-3D (125). However, more character states or multiple independent alphabets will also increase the computational resources needed.

Through the ProstT5 large language model, it is now possible to predict 3Di characters directly from amino acid sequences, rapidly speeding up this process (126). This is particularly appealing for viruses containing long open reading frames and polyproteins, which are cumbersome to conventionally predict structures for. However, the current ProstT5 language model has not been trained on polyproteins or sequences longer than 512 residues. Furthermore, additional prediction noise is added, as the same amino acid sequence can result in slightly different 3Di sequences, and care should be taken comparing 3Di characters generated from language models to those derived from AlphaFold structures (108, 126).

## OUTSTANDING QUESTIONS AND FUTURE DIRECTIONS

The rapid rate at which viruses evolve means we are left with few genomic traces intact over deep timescales. This feature of viruses has long posed challenges to reconstructing their origins and diversification. However, recent advancements in computational biology, metagenomics, and protein structure prediction have opened up new avenues, allowing us to peer back further and with greater clarity than ever before. In turn, this has begun to reshape our understanding of virus evolution, particularly at deeper timescales. Despite these advances, many fundamental questions about deep viral evolutionary history remain. We now outline some of the opportunities and challenges for the field.

## RESOLVING THE TIMESCALE AND ORIGINS OF ESTABLISHED VIRAL DISEASE

Even for some of the most well-researched viruses (e.g., HBV and hepatitis C virus), we still lack a clear understanding of how, when, and from where they have emerged in humans. Through uncovering the origins and timescale with which these viruses have emerged, we gain insight into pathways of cross-species transmission, drivers of emergence, and reservoirs of viral disease. Future efforts in this field should consider the TDR phenomenon and its effects on accurate timescale inference and integration with geographical or historical data. Models that accommodate TDR effects, such as PoW models, have shown promise (20, 127), although several improvements could be made. Existing TDR models may be compromised by substantial rate variation among lineages at deep timescales. Therefore, more realistic models should consider temporal rate shifts (Fig. 1D) and among-lineage rate heterogeneity (Fig. 1E). In addition, current approaches have been largely restricted to nucleotide sequence data. Incorporating the more conserved amino acid and structural information will provide additional evolutionary signals, crucial for resolving deeper phylogenetic relationships.

## VIRUS CLASSIFICATION AND TAXONOMY

The hierarchical classification systems with taxonomic ranks underpin our understanding and ability to communicate viral deep evolutionary history. Virus taxonomy is governed by the International Committee on Taxonomy of Viruses (ICTV). Metagenomic virus discovery studies have resulted in the discovery of a large number of highly diverse virus sequences leading to a shift in the information required for the ratification of new species from biochemical properties, morphology, replication cycle, and the virus’s host range, to one that is predominantly based on the evolutionary relationships between genetic sequences (128). The ICTV currently classifies viruses into a 15-rank taxonomic framework all the way up to the virus realm. Given the addition of structural information seems particularly useful at resolving ambiguous splits in phylogenies (108), it has clear applications for challenging groups such as the *Lenarviricota* (129). Structural phylogenetics of the *Flaviviridae* RdRp was recently used to support the split of the family into three (48), and tools that make use of structural distances to taxonomically classify virus sequences have recently been developed (130). It is not yet clear whether structural evolutionary information will be useful for individual species demarcation or whether it is best applied only for the demarcation of higher taxonomic groups (e.g., family, order, and phylum). Likewise, whether this would be in the form of phylogenetics or distance criteria is yet to be seen.

## VIRAL ORIGINS AND EARLY DIVERSIFICATION

Perhaps the most long-standing question is the origin of viruses and their proteins. Historically, three core hypotheses have been proposed (reviewed in reference 131). While still highly debated (132), it appears that the two key functional modules of viruses, those used in genome replication and virion formation, cannot be easily explained by a single scenario. Instead, a combination of scenarios likely occurred with genome replication modules arising from primordial replicons and capsid genes acquired repeatedly from cellular life (131).

We foresee several ways in which the emerging approaches discussed in this review may contribute to our understanding of viral origins. Given the speed and sensitivity of modern structural comparison methods and the rapidly increasing diversity of structural databases, large surveys to identify homologs of virus proteins are now possible. In fact, during the time of writing, the structures of about 27,000 representative viral proteins predicted using AlphaFold2 have recently been made available in the Viral AlphaFold Database, in addition to those present in the previously discussed virus structure databases. These have revealed shared folds across viruses infecting bacteria, archaea, and eukaryotes (133). Confirming that there are no closely related members of the RdRp and reverse transcriptase replication modules in cellular organisms will be key to assessing the “primordial virus world” hypothesis. In addition, cellular ancestors have not been identified for all viral capsids (119). Mapping the distribution of capsids across viruses and cellular life may allow us to better understand the source and the timing of their acquisition by viruses. Likewise, the directionality of capsid evolution—whether from cells to viruses or vice versa—could be uncovered.

More broadly, it will likely be feasible to examine the early diversification of viruses and how they have co-opted cellular proteins to expand functional space. While sequence similarity between distant viruses and between viruses to cellular proteins is frequently detected, for a large proportion of viral proteins, there are no detectable homologs and, subsequently, little is known about their functions (78). The use of structural information to annotate virus sequences lacking detectable homologs has proven useful (44, 75, 78, 134–136). For example, the origins of 14 previously undescribed orthopoxvirus proteins were demonstrated using structural prediction and search-based methods, providing evidence for the exaptation of cellular host enzymes for virus structural roles (136). As with the question about viral origins, determining the timescale and directionality of such ancient events will likely require structural comparisons and phylogenetics. Such studies could reveal fundamental aspects of virus biology, such as the determinants of viral host range and the mechanisms and fluctuations of virus genome size. However, it will undoubtedly be challenging to construct meaningful multiple sequence or structural alignments and phylogenies at this evolutionary scale.

How have viruses evolved alongside vertebrates as they diversified? Many RNA virus lineages span the breadth of vertebrates (137). For example, members of the *Coronaviridae* have been identified in all vertebrate classes, including jawless fish (subfamily *Letovirinae*) (138, 139). RdRp phylogenies of these viruses largely mirror those of their hosts, suggesting virus-host co-divergence over millions of years, likely beginning in aquatic vertebrates and following their transition onto land (138). Yet, key violations of patterns of virus-host co-divergence are present. For example, lamprey-associated letoviruses are phylogenetically positioned within the bony fish clade rather than falling basal to bony fish, contradicting host taxonomy (138). Disentangling patterns of co-divergence and cross-species transmission over the diversity of viruses across vertebrate life is challenging, especially at deep evolutionary timescales. Topological comparisons of virus and host trees have been used to infer co-divergence. While of benefit, it is often difficult to distinguish cophylogenetic signal from phylogenetic congruence, which may be a result of other factors such as preferential host switching, where viruses are preferentially transferred to closely related hosts (140). Such methods may wrongly infer time-inconsistent host switches between non-contemporary host lineages (141, 142). Time-calibrated phylogenies provide a clear framework to test co-divergence scenarios and place the predicted events into a broader temporal and geological context (143). Building on this, explicit modeling of co-divergence in a Bayesian framework appears promising (144, 145) and could allow for the joint inference of evolutionary, geographical, and structural information while also accounting for rate variation and saturation.
